# Metformin Reduces Repeat Mild Concussive Injury Pathophysiology

**DOI:** 10.1523/ENEURO.0421-21.2021

**Published:** 2022-01-05

**Authors:** Erica L. Underwood, John B. Redell, Mark E. Maynard, Nobuhide Kobori, Michael J. Hylin, Kimberly N. Hood, Rebecca K. West, Jing Zhao, Anthony N. Moore, Pramod K. Dash

**Affiliations:** Department of Neurobiology and Anatomy, The University of Texas McGovern Medical School, Houston, TX 77030

**Keywords:** axonal injury, cognitive dysfunction, mild traumatic brain injury, oxygen consumption rate, repeat concussion, tissue respiration

## Abstract

Mild traumatic brain injury (mTBI) can initiate complex pathophysiological changes in the brain. Numerous cellular and molecular mechanisms underlying these pathologic changes are altered after injury, including those involved in energy utilization, excitotoxicity, ionic disturbances, and inflammation. It is thought that targeting multiple mechanisms may be necessary to produce meaningful reductions in brain pathology and to improve outcome. Previous studies have reported that the anti-diabetic drug metformin can also affect inflammatory, cell survival, and metabolic outcomes, possibly by acting on multiple targets and/or pathways. We therefore questioned whether metformin treatment can reduce pathology after repeat mild closed head injury (rmCHI) in male C57Bl/6 mice. We found that metformin, administered acutely after each head impact, resulted in markedly reduced white matter damage, astrogliosis, loss of hippocampal parvalbumin neurons, and improved mitochondrial function. In addition, both motor and cognitive functions were significantly improved when tested after discontinuation of the treatment. These studies suggest that metformin may be beneficial as a treatment for repeat concussion.

## Significance Statement

Repeat concussions (or repeat mild traumatic brain injury; rmTBI) can occur in persons participating in contact sports, and in military personnel. Unfortunately, there are no approved treatments to lessen the consequences of rmTBI. It is thought that outcome from rmTBI is influenced by several secondary events, including altered brain metabolism, inflammation, and damage to brain cells. Here, we show that the anti-diabetes drug metformin reduces repeat concussion brain pathology and improves motor and cognitive functions.

## Introduction

Both clinical and experimental studies have shown that mild traumatic brain injury (mTBI; often referred to as concussion) can cause cognitive (e.g., learning and memory) and behavioral (e.g., anxiety and depression) impairments in the absence of overt brain damage (Jenkins et al., 1989; Lyeth et al., 1990; Gurkoff et al., 2006; Choe et al., 2012; Shenton et al., 2012; Barkhoudarian et al., 2016; Meconi et al., 2018). In the majority of cases, the cognitive and behavioral impairments do not persist, and resolve within days to weeks after a single concussive event (Capruso and Levin, 1992; McCauley et al., 2014). In contrast, persons who sustain repeated mTBI (rmTBI) often present with prolonged cognitive and behavioral symptoms that, in some cases, can last months-to-years after injury. Although the cellular and molecular mechanisms that underlie these symptoms are still incompletely understood, it is thought that multiple mechanisms, including white matter damage, astrogliosis, neuroinflammation, and mitochondria dysfunction, are involved. There is a growing consensus that interventions that target multiple pathologic mechanisms may be necessary to provide a meaningful improvement in patient outcomes (Saatman et al., 2008; Margulies et al., 2016).

Metformin (1,1-dimethylbiguanide hydrochloride) is a widely prescribed medication for type 2 diabetes. Although its mechanism(s) of action is not fully understood, current evidence suggests that metformin may target multiple signaling pathways to exert the numerous beneficial effects that have been reported in a variety of model systems (Viollet et al., 2012; Alimoradi et al., 2021; Kaneto et al., 2021). Some of the signaling pathways affected by metformin have implications for mitigating brain injury pathophysiology. For example, metformin can regulate cellular energy metabolism by activating AMP-activated protein kinase (AMPK), an important intracellular energy sensor that is deactivated following TBI (Hill et al., 2016). Metformin can also influence cellular energy balance and metabolism independently of the AMPK pathway by regulating mTOR (Kalender et al., 2010; Ben Sahra et al., 2011; Do et al., 2013). Inhibition of the expression and activation of the proinflammatory transcription factor nuclear factor κB (NF-ĸB) may contribute to the many anti-inflammatory activity reports of metformin (Oliveira et al., 2016; Ou et al., 2018; Inayat et al., 2019; Kanigur Sultuybek et al., 2019), and increased cellular survival after an insult has been attributed to the metformin-mediated increase in phosphorylation of the pro-survival/anti-apoptotic kinase Akt (Ge et al., 2017; Tang et al., 2017). Metformin can also alter expression of a number of miRNAs involved in various cellular processes (Alimoradi et al., 2021), and alter the gut microbiome (Wu et al., 2017). Although metformin has been shown to reduce cell loss and improve cognitive outcome following moderate-to-severe TBI, it has not been examined whether metformin can reduce the pathophysiology of rmTBI in which overt damage is not observed (Hill et al., 2016; Tao et al., 2018; Taheri et al., 2019; Rahimi et al., 2020).

By virtue of its impact on inflammation, growth, cell survival, and metabolic signaling cascades, we hypothesized that metformin would be efficacious in reducing rmCHI-related pathologies. To test this, we administered metformin to rmCHI animals 30 min after each injury (once a day for four consecutive days). Our results show that daily postinjury metformin administration reduced axonal damage, reduced inhibitory neuron loss, and enhanced ATP-linked respiration in the hippocampi of rmCHI mice. Furthermore, motor and memory dysfunction were significantly reduced in rmCHI animals that received metformin. These results suggest that further studies may be warranted to determine whether metformin treatment after an mTBI may be beneficial in a clinical setting.

## Materials and Methods

### Animals

C57Bl/6 male mice (15 weeks of age) were single housed on a 12/12 h light/dark cycle, with *ad libitum* access to food and water. Experiments were performed during the light cycle. As C57BL/6 male mice are separated by the vendor at 12 weeks of age (The Jackson Laboratory), mice were individually housed. All experimental procedures were conducted in accordance with the *Guide for the Care and Use of Laboratory Animals* of the National Institutes of Health, and approved by the UTHealth McGovern Medical School Animal Care and Use Committee.

### Repeat closed head injury (CHI)

Mild CHIs (mCHIs) were delivered essentially as have been described previously (Mouzon et al., 2012; Hylin et al., 2013; Maynard et al., 2020). Male C57BL/6 mice (20–25 g) were initially anesthetized with 5% isoflurane in a 1:1 O_2_/N_2_O mixture, mounted on a stereotaxic frame, and anesthesia was then maintained with a 2.5% isoflurane and 1:1 O_2_/N_2_O mixture via a face mask. A midline incision was made and the soft tissue reflected to expose the skull. Anesthesia was discontinued, and the mouse was quickly transferred to a foam pad and positioned to make the head level with the body. At 40 s after discontinuation of anesthesia (a time point at which mice have typically regained their tail withdrawal reflex), a single impact was applied to the skull using a metal impactor tip (5 mm in diameter) driven at a velocity of 5.0 m/s to a depth of 1.0 mm. The center of the tip was located midway between λ and bregma, over the sagittal suture. Immediately following the injury, the animals were monitored for apnea, and when normal breathing was observed, the scalp was closed using sterile surgical staples. Animals with obvious skull fractures (∼15%) were excluded from this study. Mice received one injury/day for four consecutive days. Sham mice received daily anesthesia but were not injured.

### Metformin administration

Metformin hydrochloride was dissolved in sterile saline at a concentration of 50 mg/ml. Metformin was injected intraperitoneally at a dose of 250 mg/kg 30 min after each CHI. Vehicle controls received an equal volume of sterile saline (100 μl), injected 30 min after each injury. As a number of studies have examined the effects of metformin in naive animals, metformin was not administered to our sham controls to minimize the number of animals used in this study (Potter et al., 2010; Pintana et al., 2012; Li et al., 2019; Thinnes et al., 2021).

### Measurement of plasma and brain metformin concentration

For determining plasma and brain metformin concentrations, uninjured mice were intraperitoneally injected with 250 mg/kg metformin. Metformin has been reported to have plasma half-life of 2–6 h in mice (Kinaan et al., 2015). Six hours after injection, animals were euthanized and blood was collected. Their brains were then removed, and hippocampal, cortical, and cerebellar tissues dissected, weighed, and homogenized in water at 0.3 mg/ml (w/v). Methanol (400 μl) and acetonitrile (500 μl) were added to 100 μl homogenate, vortexed 60 s, then incubated for 10 min at room temperature (RT). The solution was centrifuged at 20,000 × *g* for 10 min at RT, and the supernatant removed and passed through a 0.45 μm nylon syringe filter. The solution was dried at 45°C in a vacuum centrifuge, and resuspended in 120 μl mobile phase (see below). Chromatography was conducted using a Shimadzu Nexera UPLC with dual LC-30AD pumps and an integrated Prominence degasser (DGU-20A). The mobile phase consisted of a 90:10 mixture of 0.15 m ammonium acetate (pH 5.5) and acetonitrile filtered through a 0.22 μm nylon membrane. Separation was conducted on a Discovery HS F5-5 (15 cm × 4.6 mm, 5 μm) column at a rate of 1 ml/min. Detection of metformin (40 μl injection volume) was conducted using a Prominence UV/VIS detector (SPD-20A) at 236 nm. For analysis of metformin in plasma, 100 μl was deproteinized with the methanol:acetonitrile as described above. A standard curve was prepared by spiking naive extracts (100 μl each) with a known amount of metformin (0.23, 0.47, 0.94, 1.88, 3.75, 7.5, or 15 nmol), processed as described above, and subsequently used to generate a standard curve that was used to calculate the concentration of metformin in the experimental samples. Spike-in assays revealed that the recovery from tissue and serum were 95% and 79%, respectively. Calculations were corrected based on recovery.

### Immunohistochemistry

Mice were deeply anesthetized using sodium pentobarbital (100 mg/kg). Once the animal failed to respond to foot and tail pinch, the animal was transcardially perfused with PBS followed by 4% paraformaldehyde in PBS. Brains were removed, postfixed overnight in perfusant, then cryoprotected in a 30% sucrose solution in PBS. Coronal brain sections (40 μm) were prepared using a cryostat. Sections containing the dorsal hippocampus (bregma −1.58 to −2.30 mm) were used for analysis. Immunohistochemistry was performed by incubating brain sections with primary antibodies (1 μg/ml) overnight at 4°C in a solution consisting of PBS with 0.25% Triton X-100 (PBST), 2.0% BSA, and 2.5% normal goat or horse serum. After extensive washing in PBST, tissue sections were incubated for 1 h in species-specific secondary antibodies conjugated to Alexa Fluors. Sections were washed in PBST, mounted on microscope slides, coverslipped with Fluoromount G (Thermo Fisher Scientific), and visualized using epifluorescence detection. Images were captured using a Retiga 6000 camera with settings that remained consistent across groups. Immunoreactivity in the hippocampus was quantified using ImageJ by carefully outlining the hippocampus on three sections/animal and determining mean fluorescence signal intensity. The corpus callosum and hippocampal commissure (±0.5 mm from midline) were combined for analysis.

### Silver staining

Silver staining was conducted on free-floating sections using a kit from FD Neurotechnologies following the vendor instructions. Processed sections were mounted to glass slides, clarified using xylene, and coverslipped with Fluoromount G (Thermo Fisher Scientific). Slides were examined using an inverted microscope with bright-field capabilities. Images were captured using settings that remained consistent across groups. The corpus callosum and hippocampal commissure (±0.5 mm from midline) were outlined on three sections/animal using ImageJ, and the calculated mean optical densities averaged and used for comparison across groups.

### Capillary westerns and analysis

Mice were euthanized and hippocampal tissues dissected under ice cold artificial CSF (aCSF; 120 mm NaCl, 3.5 mm KCl, 1.3 CaCl_2_ mM, 1 mm MgCl_2_, 0.4 mm KH_2_PO_4_, 5 mm HEPES, and 10 mm d-glucose; pH 7.4) and snap frozen on dry ice. Total protein homogenates were prepared using a Potter–Elvehjem homogenizer in lysis buffer containing 10 mm Tris-HCl, pH 7.4, 1 mm EDTA, 1 mm EGTA, 1 mm Na_3_VO_4_, 5 mm NaF, 5 mm Na_2_MoO_4_, 1 mm DTT, and 1× protease and phosphatase inhibitor cocktails (Thermo Fisher Scientific). Total protein concentration was measured by BCA assay using BSA as the reference standard. Sample aliquots were diluted into 1× sample buffer and protein content equalized. Target proteins were quantified using an automated capillary immunoassay system (Wes system; Protein Simple). Immunoreactivity was detected with a luminol-peroxide solution and a series of timed exposures were collected. The images were analyzed using Compass for SW (version 3.1.7; Protein Simple). Results were normalized against β-actin and presented as percent control.

### Tissue respiration

Respiration measurements of brain biopsy punches were performed using an XF96 Extracellular Flux Analyzer (Seahorse Bioscience). At the indicated time points after injury, brains were rapidly removed and immersed in ice-cold (4°C–5°C) aCSF that had been oxygenated for 1 h with 95% O_2_:5% CO_2_. Coronal sections (225 μm) were cut using a McIlwain tissue chopper (Ted Pella. Inc.), then transferred to a holding chamber containing room temperature (∼23°C) continuously oxygenated aCSF and incubated for at least 30 min. Before making tissue punches from the structures of interest, sections were individually transferred to a biopsy chamber containing freshly oxygenated aCSF, then 0.5-mm diameter punches were excised using a stainless-steel biopsy punch needle (WellTech Rapid-Core). Punches were ejected directly into a XF96 Cell Culture Microplate (101085–004; Seahorse Bioscience) containing 100-μl room temperature assay media (aCSF supplemented with 4 mg/ml BSA and 0.6 mm pyruvate). After loading all samples, the microplate was incubated in a CO_2_-free incubator at 37°C for 15 min, followed by addition of 80 μl/well of 37°C assay medium (bringing the total volume to 180 μl/well). Punches were placed in the center of the measurement area of each well and the plate was returned to the incubator. Assay drugs were loaded into the Seahorse XFe96 Extracellular Flux Assay kit as indicated below. Drugs were prepared at 10× working concentrations in oxygenated aCSF (pH 7.4) and delivered sequentially to achieve final concentrations of port A: oligomycin (25 μg/ml), port B: FCCP + pyruvate (7.5 μm and 7.7 mm, respectively), and port C: antimycin A + rotenone (10 μm and 5 μm, respectively). The dose of each of these reagents was based on optimization experiments to achieve maximum inhibition. The duration of sampling time [for calculating the oxygen consumption rate (OCR)] for each condition was determined to allow the effect of each drug to reach a steady state. Wells with low basal activity (<20 pmol/min OCR), and/or failing to respond to FCCP/pyruvate, were excluded from analysis.

### Mitochondria isolation and respiration measurement

Mitochondria from brain tissues were isolated using Percoll density gradient centrifugation as previously described (Sims and Anderson, 2008; Sekine et al., 2015). Hippocampi were rapidly removed while submerged under ice-cold aCSF, rinsed with cold isolation buffer (10 mm EDTA, 100 mm Tris, and 12% Percoll solution; pH 7.4), and homogenized in 3 ml isolation buffer using a Dounce homogenizer (pestle A: eight strokes; pestle B: eight strokes). A small fraction of each homogenate was removed for determining protein concentration. The remaining homogenate was then layered onto a discontinuous Percoll gradient (26% and 40% Percoll) and centrifuged at 4°C for 10 min at 30,700 × *g*. The enriched mitochondrial fraction was removed from the 26%:40% interface, transferred to individual centrifuge tubes, and diluted 1:4 with isolation buffer. Mitochondria were then pelletized by centrifugation (16,700 × *g* at 4°C) for 10 min. The supernatant was discarded and the pellet suspended in mitochondrial assay solution (MAS; 70 mm sucrose, 220 mm mannitol, 10 mm KH_2_PO_4_, 5 mm MgCl_2_, 2 mm HEPES, and 1 mm EGTA) with substrates (10 mm pyruvate and 5 mm malate) and 0.2% BSA. An equal volume of suspension (containing 1 μg of mitochondrial protein) was dispensed into individual wells of an Agilent XF96 cell culture microplate and centrifuged (2000 × *g* at 4°C) for 30 min. Assay medium was added to the wells containing mitochondria to bring the final volume to 180 μl before the plate being incubated at 37°C for 30 min and transferred to the analyzer for analysis. For assessing the various aspects of mitochondrial respiration, inhibitor/uncoupler stocks were loaded into the drug ports of a hydrated sensor cartridge in the following order: (1) oligomycin (2.5 μg/ml final), (2) FCCP (4 μm final), and (3) antimycin A (4 μm final) + rotenone (2 μm final). The assay protocol consisted of a minimum of three cycles of OCR measurements for each measurement period. Each cycle consisted of a 2-min “mix” period and 2-min “wait” period, followed by a 3-min “measure” period.

### Vestibulomotor and motor functions

Foot-fault and beam balance tasks were used to determine an animal’s postinjury vestibulomotor and motor performance on three consecutive days, beginning 24 h after the last injury as described previously (Varma et al., 2002; Tehranian et al., 2008). Foot-fault was evaluated by placing the animal on a wire grid (1 × 1 cm) and the number of foot misplacements out of a total of 50 steps were counted. A fault was defined as when a front paw missed and appeared below the plane of the wire grid. For the beam balance procedure, the animal was placed on the stationary beam for a period of 60 s. A trial ended, and the time was recorded, when the animal either fell off the beam, or 60 s had elapsed. Foot-fault and beam balance tests were repeated three times each day to give an average daily score.

### Novel objection recognition (NOR)

Animals were habituated to the empty training chamber (30 × 30 cm) for two 10-min periods per day for 2 d. On the third day, training was conducted by placing two identical objects in the box, and the animal was allowed to freely explore for 10 min. Twenty-four hours later, the animal was tested for its long-term memory by replacing one of the objects in the chamber with a new object of a different shape, and allowed to again freely explore the objects for 10 min. Trials were video recorded and the time spent exploring each object during the familiarization period and the memory test was tracked by an experimenter blinded to group designation. To eliminate potential object biases, the objects used in the familiarization and testing phases were previously verified as being equally interesting to naive mice. The choice of familiar and novel objects, and their placement in the chamber, was randomized across animals.

### Context discrimination

Context discrimination was conducted by first preexposing animals for 10 min to two different contexts. These contexts shared certain features in common (background noise, horizontal grid floor, animal handling to and from the room), but differed in others (cues, floor color, shape, and scent). Animals were given two 3-min preexposure trials (one trial in each of the two different contexts). After the preexposure, one context was designated as the “shock” cage in which animals received a 2 s, 1.0 mA shock at the end of the 3-min trial. The other context was designated as the “safe” cage in which no shock was administered. The designation of the “safe” versus “shock” context, and the exposure order (e.g., safe then shock, or shock then safe), was randomized across animals. Twenty-four hours later, discrimination between the safe and shock contexts was assessed by monitoring freezing behavior while the animal explored each of the two chambers (3 min each). The difference in freezing time in the “shock” versus “safe” cage was used as a measure of context fear discrimination.

### Experimental design and statistical analyses

The number of animals used for each analysis are listed in the corresponding result section. Replication of biochemical and histologic data were conducted as described for each technique in the applicable Materials and Methods section. Data were statistically analyzed using Sigma Plot 12.0. All data were subjected to a Shapiro–Wilk test for normality and a Spearman rank correlation for equal variance before statistical comparison. For respiration measurements, a one-way ANOVA was used to compare across groups at a single time point. Foot fault, beam balance and context fear discrimination were analyzed across days and groups using two-way repeated measures ANOVAs. Both group main and interactions of group and time were considered indicators of group differences. Where applicable, Bonferroni-corrected *post hoc* comparisons were made. For the NOR task, the percent of time spent with the novel compared with familiar object was analyzed within groups using paired, two-tailed Student’s *t* tests. All data are reported as mean ± SEM, and *p* values were deemed statistically significant if *p* < 0.05.

## Results

### Metformin and axonal pathologies after rmCHI

**Figure 1.**
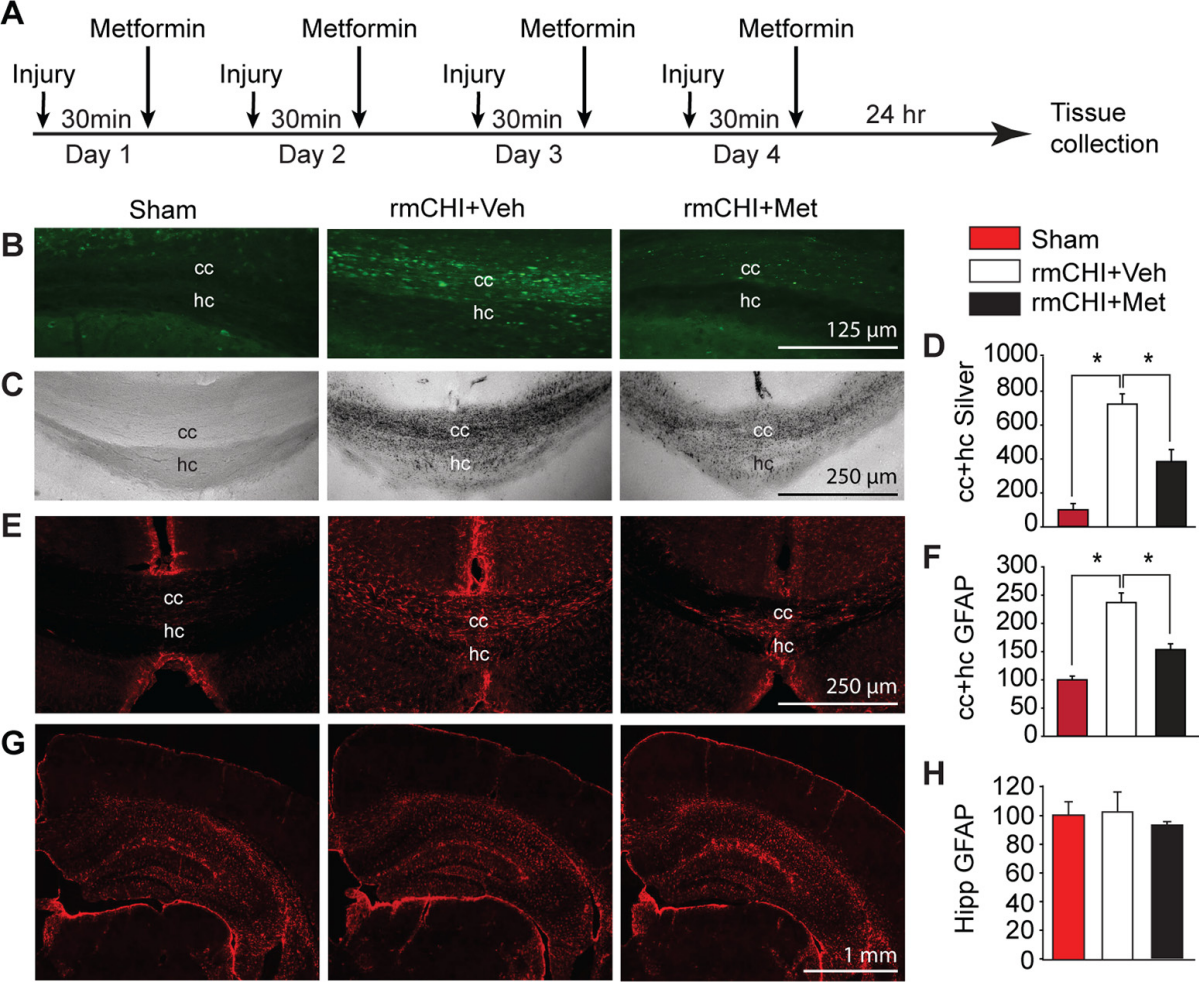
Postinjury metformin administration reduced rmCHI-triggered axonal damage and astrogliosis. ***A***, Schematic showing the injury and drug treatment paradigm. Representative photomicrographs showing (***B***) APP accumulation and (***C***) enhanced silver impregnation in the corpus callosum (cc) and hippocampal commissure (hc) of a sham, a rmCHI+Veh, and a (rmCHI+Met) mouse. ***D***, Summary graph showing that rmCHI significantly increased silver impregnation in the cc and hc, which was reduced in animals treated with metformin. ***E***, Representative images of glial fibrillary acidic protein (GFAP) immunoreactivity showing increased reactive astrogliosis in the cc and hc of a rmCHI+Veh mouse, and reduced staining in injured a rm+Met mouse, as compared with sham. ***F***, Summary data for GFAP immunoreactivity in the cc+hc fiber tracts. ***G***, Images of hippocampi and parietal cortex from a sham, an rmCHI+Veh mouse, and an rmCHI+Met mouse demonstrating GFAP immunoreactivity. ***H***, Summary data for GFAP immunoreactivity in the hippocampus. Data are presented as the mean ± SEM; *p < 0.05 by one-way ANOVA.

The timeline for injury, drug administration, and tissue collection is shown in Figure 1*A*. Metformin (250 mg/kg) or an equal volume of vehicle was administered via intraperitoneal injection 30 min after each injury. This dose was based on previous studies that used metformin in mice (Lengyel et al., 2015; Hill et al., 2016; Karise et al., 2019; Wang et al., 2020a). After correction for body surface area (Nair and Jacob, 2016), this dose is equivalent to a 23 mg/kg/d dose in humans, consistent with the guidance for dosing in type 2 diabetes (1500–2500 mg/d for an 80-kg person).

Previous clinical and experimental studies have reported that mild TBI causes damage to major white matter tracts in the brain (Bennett et al., 2012; Bramlett and Dietrich, 2015; Palacios et al., 2020), including the corpus callosum. To examine whether metformin reduces corpus callosum damage, tissue sections from sham, rmCHI mice + 250 mg/kg metformin, and rmCHI+Veh (30 min after each injury) were examined for APP immunoreactivity, an indicator of axonal damage, 24 h after the last injury (Stone et al., 2004; Marmarou et al., 2005). Figure 1*B* shows representative photomicrographs of APP immunoreactivity within the corpus callosum/hippocampal commissure region in tissue sections from a sham, an rmCHI mouse-treated with vehicle, and an rmCHI mouse treated with metformin. Numerous APP-immunopositive axons were detected in the corpus callosum and hippocampal commissure in the rmCHI+Veh animals. APP immunoreactivity was markedly reduced in animals that received the postinjury metformin treatment, suggesting reduced axonal injury. We then performed silver staining to further examine the extent of axonal injury in these fiber tracts. The representative photomicrographs of silver-stained brain sections presented in Figure 1*C* show that, consistent with the accumulation of APP, rmCHI resulted in enhanced silver impregnation in the corpus callosum and hippocampal commissure, while staining was reduced in rmCHI animals acutely treated with metformin (Fig. 1*C*). Quantification (*n* = 4/group) of silver impregnation within these white matter tracts showed there was a significant increase in axonal damage after rmCHI (*F = *22.806, *p *<* *0.001) that was significantly attenuated by metformin treatment (Fig. 1*D*).

Astrocytes are activated in response to injury and are thought to contribute to evolving brain pathology by taking up extracellular glutamate, regulating inflammation, and forming a glial scar that can impede regeneration of damaged axons (Floyd and Lyeth, 2007; Burda et al., 2016). Increased GFAP immunoreactivity (an indicator of activated astrocytes) can be seen in the corpus callosum and hippocampal commissure of injured animals 24 h after the last injury, indicating astrocyte activation (Fig. 1*E*). Quantification (*n* = 4/group) of GFAP immunoreactivity in the corpus callosum and hippocampal commissure revealed that the injury-triggered increase in GFAP immunoreactivity was significantly reduced by acute postinjury metformin treatment (*F = *32.925, *p *<* *0.001; Fig. 1*F*). Increased GFAP immunoreactivity was predominately observed in white matter tracts as GFAP immunoreactivity was not altered in either the hippocampus or injured cortex (Fig. 1*G*,*H*).

### rmCHI-triggered loss of hippocampal parvalbumin-positive inhibitory neurons and metformin treatment

**Figure 2.**
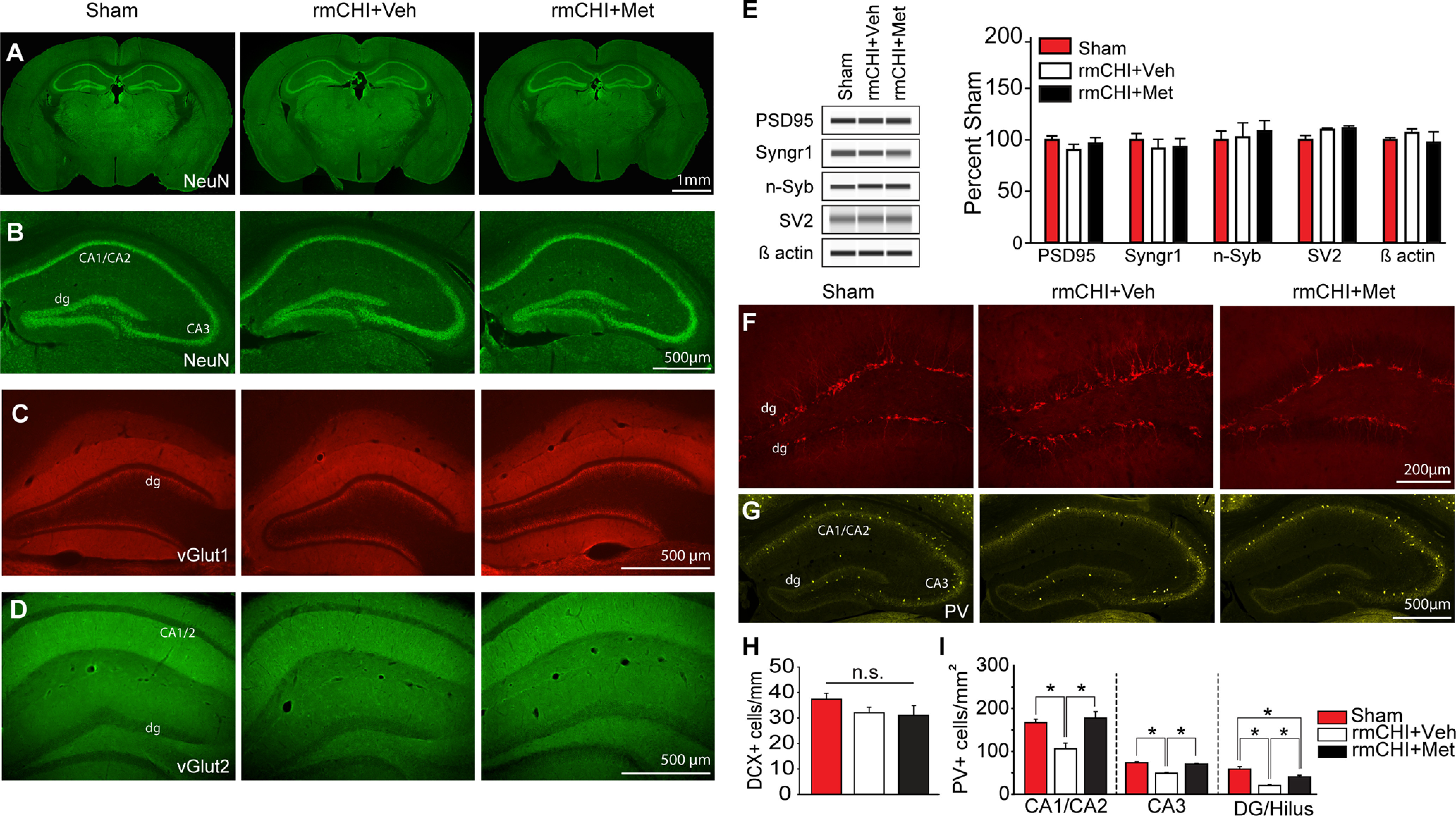
Metformin treatment preserves hippocampal parvalbumin-expressing cells after rmCHI. ***A***, Montage images of coronal mouse brain sections and hippocampi from sham, two-week postinjury rmCHI mouse treated after each injury with vehicle (rmCHI+Veh), and a two-week postinjury rmCHI mouse treated after each injury with metformin (rmCHI+Met). Sections were immunostained with antibodies against (***A***, ***B***) the neuronal marker NeuN, (***C***) the synaptic vesicle protein Vesicular glutamate transporter 1 (vGlut1), and (***D***) the synaptic vesicle protein Vesicular glutamate transporter 2 (vGlut2). ***E***, Representative capillary western analyses and summary data showing that neither rmCHI nor postinjury metformin treatment had any demonstrable effect on the hippocampal levels of postsynaptic density protein 95 (PSD95), synaptogyrin 1 (Syngr1), neuronal synaptobrevin (n-Syb), or synaptic vesicle protein 2 (SV2). Representative photomicrographs showing (***F***) doublecortin (DCX) and (***G***) parvalbumin (PV) immunoreactivity in the hippocampus of sham, rmCHI+Veh, and rmCHI+Met mice. ***H***, Summary data (*n* = 4/group) showing that rmCHI had no statistical effect on the number of doublecortin-positive newborn neurons in the hippocampus when examined 3 d after injury. ***I***, Summary data (*n* = 3/group) showing that rmCHI reduced the number of PV-positive interneurons in all hippocampal regions examined. Metformin treatment reversed the loss of PV-positive interneurons in the CA1, CA2, and CA3 regions, and attenuated the loos in the DG/hilus. dg: dentate gyrus; *p < 0.05 by one-way ANOVA.

Consistent with the classification of the injury as mild, when tissue sections collected two weeks after rmCHI were immunostained for NeuN, no overt neuronal loss or tissue damage was observed (Fig. 2*A*). Higher magnification images of the hippocampus show that rmCHI did not cause visible neuronal loss nor disruption of the neuronal layers in either rmCHI mice treated with vehicle, or rmCHI mice treated with metformin, as compared with sham controls (Fig. 2*B*). Furthermore, no changes in the immunoreactivity for the synaptic proteins vGlut1 (Fig. 2*C*) or vGlut2 (Fig. 2*D*) were observed. Capillary western analysis (*n* = 4/group) of hippocampal total protein extracts showed no significant changes in the immunoreactivities for postsynaptic density protein 95 (PSD95), synaptogyrin 1 (Syngr1), neuronal synaptobrevin (n-Syb), or synaptic vesicle protein 2 (SV2; Fig. 2*E*), in contrast to what has been observed after higher magnitudes of TBI (Ansari et al., 2008; Sen et al., 2017). Lastly, we detected no change in the number of doublecortin-positive newborn hippocampal neurons (examined 72 h after the last injury; *n* = 5/group) as a result of repeat injury, or in response to metformin treatment after injury (Fig. 2*F*,*H*).

It has been suggested that inhibitory neurons, particularly parvalbumin-positive fast-spiking interneurons, are vulnerable to all magnitudes of TBI (Hsieh et al., 2017; Tucker et al., 2019; Broussard et al., 2020; Cantu et al., 2020; Carron et al., 2020). rmCHI has been demonstrated to result in the loss of hippocampal parvalbumin-positive interneurons (Tucker et al., 2019), while metformin treatment can enhance cell survival signaling in some experimental models (Ge et al., 2017; Tang et al., 2017). We therefore examined whether metformin administered post-rmCHI could mitigate parvalbumin-positive neuron loss. Figure 2*G* shows representative images of parvalbumin immunostaining in the hippocampi from a sham, a two-week post-rmCHI mouse treated with vehicle, and a two-week post-rmCHI mouse treated with 250 mg/kg metformin after each injury. The summary data presented in Figure 2*I* (*n* = 3/group) shows that a significant injury-associated decrease in the number of parvalbumin-positive cells was detectable in the CA1/CA2 subfield (*F = *9.476, *p = *0.014), the CA3 subfield (*F = *54.355, *p < *0.001), and the dentate/hilar region (*F = *22.622, *p = *0.002) two weeks after rmCHI in vehicle-treated mice. Postinjury treatment with metformin ameliorated the loss of parvalbumin immunostaining.

### Metformin and hippocampal mitochondrial function after rmCHI

**Figure 3.**
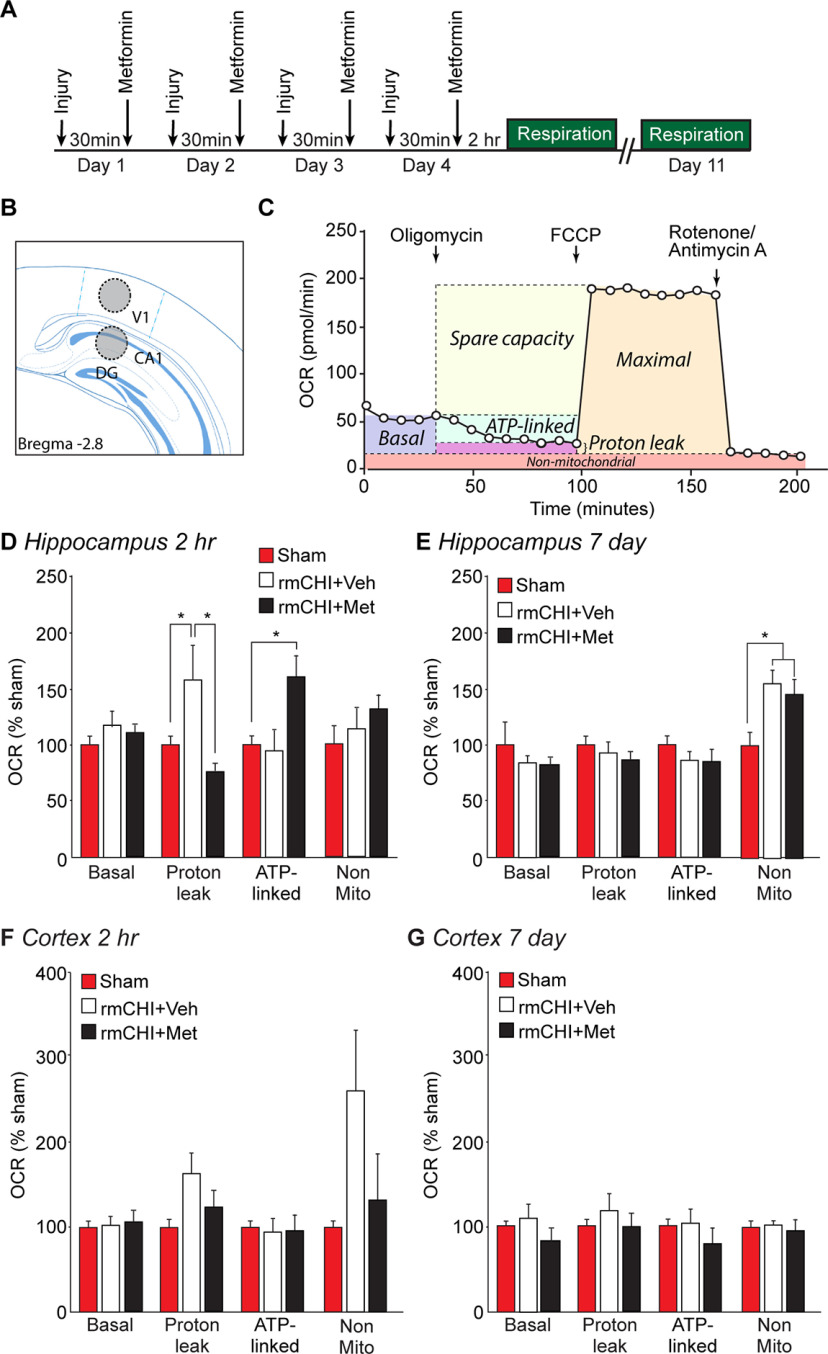
Postinjury metformin administration reduced mitochondrial proton leak and increased ATP-linked respiration in the hippocampus after rmCHI. ***A***, Timeline for injury (once daily for 4 d), metformin administration, and respiration measurements either 2 h or 7 d after the final injury was given on the fourth day. ***B***, Drawing of a coronal brain section showing the relative positions of the tissue punches taken for cortical and hippocampal tissue respiration measurements. ***C***, A representative trace of the oxygen consumption rate (OCR) showing the different phases of mitochondrial respiration that can be identified using inhibitors. Changes in OCR resulting from the addition of the various mitochondrial inhibitors/uncouplers is used to determine the different components of mitochondrial (and nonmitochondrial) respiration. Summary results showing the effect of rmCHI and metformin treatment on the various aspects of mitochondrial respiration in the hippocampus at (***D***) 2 h and (***E***) 7 d postinjury. Summary results showing the effects of rmCHI and metformin treatment on mitochondrial respiration in the cortex at (***F***) 2 h and (***G***) 7 d postinjury. Data are presented as the mean ± SEM; *p < 0.05 by one-way ANOVA.

To examine the effect of rmCHI and postinjury metformin treatment on mitochondrial respiration, we measured OCR using tissue biopsy punches. A schematic of the experimental design is shown in Figure 3*A*. Dorsal hippocampal (containing stratum oriens, CA1 stratum pyramidale, and stratum radiatum) and parietal cortex (containing layers 2–6; AP −4.52 mm, ML 4–5 mm) tissue punches were prepared from sham, rmCHI+Veh and rmCHI+Met mice (Fig. 3*B*). A representative OCR curve indicating the responses to various inhibitor injections used to interrogate different aspects of mitochondrial respiration is shown in Figure 3*C*. Basal OCR is determined by respiration measurement before any inhibitor injection. The ATP synthase inhibitor oligomycin blocks proton flux through ATP synthase (Complex V), reducing OCR to reveal respiration linked to ATP synthesis. The residual proton flux and related oxygen consumption after oligomycin addition to the reaction represents a measure of proton leak. The uncoupler FCCP, which abolishes the proton gradient, results in maximal OCR. Finally, the addition of rotinone (a Complex I inhibitor) and antimycin A (an inhibitor of cytochrome *c* reductase) abolishes mitochondrial-mediated oxygen respiration. Any remaining residual oxygen consumption after addition of rotinone/antimycin A is attributed to nonmitochondrial oxygen-consuming enzymes in the sample, such as oxidases.

The summary results (*n* = 6 animals/group; 5 punches/region) shown in Figure 3*D* indicate that rmCHI significantly increased proton leak in the dorsal hippocampus 2 h after the last injury (*F = *6.765; *p = *0.005; *post hoc* sham vs rmCHI+Veh: *p = *0.035). Proton leak was reduced by metformin treatment (rmCHI+Veh vs rmCHI+Met: *p = *0.005), and was accompanied by a significant increase in ATP-linked respiration (*F = *5.201; *p = *0.015; sham vs rmCHI+Met: *p = *0.013). Of note, these changes were not observed in mice that were euthanized 2 h after a single CHI (data not shown), and had normalized by 7 d postinjury (Fig. 3*E*). Nonmitochondrial respiration (oxygen consumption by enzymes such as monoamine oxidase, xanthine oxidase, etc.) in the hippocampus was not different 2 h after rmCHI, but was significantly enhanced 7 d after rmCHI (*F = *8.06; *p = *0.003; Fig. 5*E*). Metformin treatment did not affect this delayed increase in nonmitochondrial respiration (rmCHI+Veh vs rmCHI+Met: *p = *0.452). There were no significant changes in mitochondrial respiration detected in cortical punches taken at either 2 h (Fig. 3*F*) or 7 d (Fig. 3*G*) postinjury.

### High-dose metformin and mitochondrial Complex I

**Figure 4.**
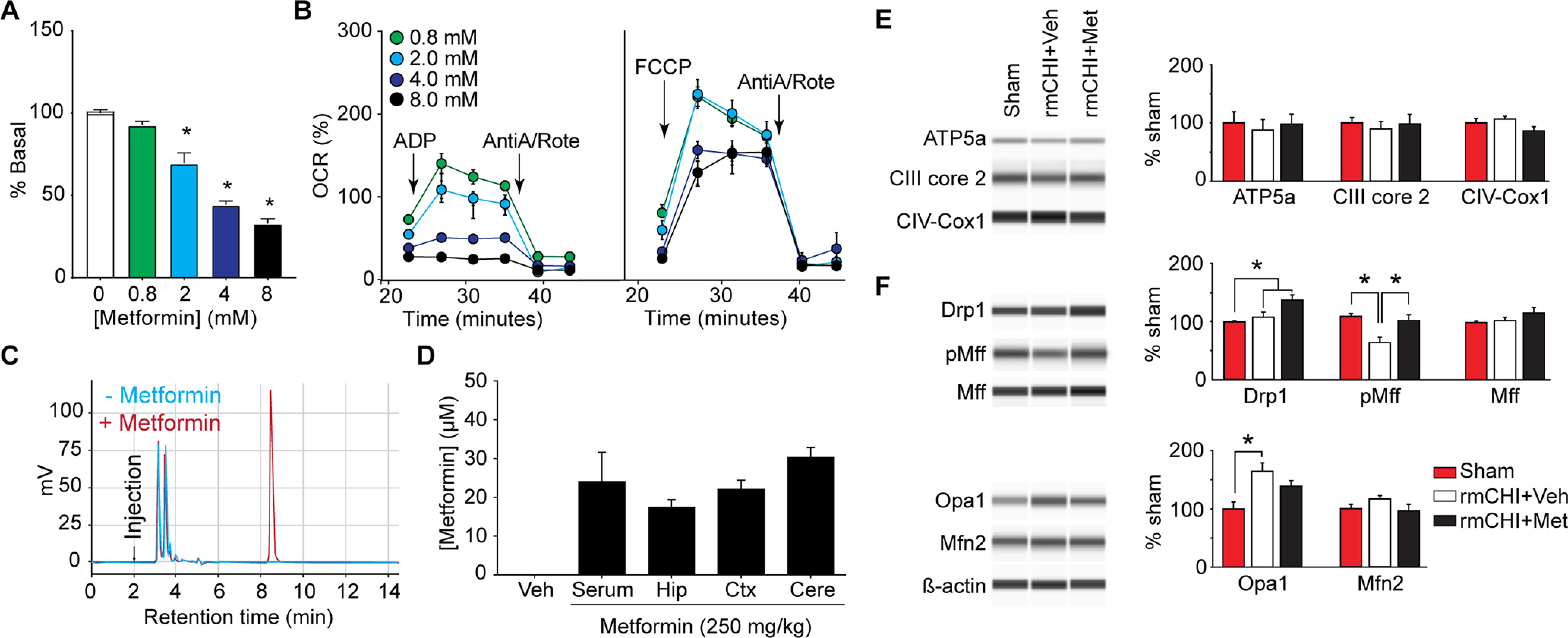
Metformin activates pathways involved in mitochondrial fission. ***A***, Summary data showing that ≥2 mm metformin present in the reaction buffer significantly inhibits basal respiration of isolated hippocampal mitochondrial; *p < 0.05 by one-way ANOVA compared with vehicle (0 mm metformin). ***B***, Addition of ADP, which requires an intact proton gradient to stimulate oxygen consumption, had no effect on mitochondria treated with ≥4 mm metformin. Trifluoromethoxy carbonylcyanide phenylhydrazone (FCCP), which uncouples oxygen consumption from ATP synthesis by disrupting the proton gradient, stimulated OCR at all metformin concentrations. ***C***, Representative HPLC chromatographs of cortical tissue extracts in the absence (blue) and presence (red) of metformin. ***D***, Summary data showing the concentration of metformin in various tissues detected 6 h after intraperitoneal administration of 250 mg/kg to mice. Data are presented as the mean ± SEM. ***E***, Representative capillary western analyses and summary data (*n* = 5/group) showing that neither rmCHI nor metformin treatment altered the expression of components of the electron transport system 2 h after the last injury. ATP5a: ATP synthase F1 subunit α; CIII core 2: cytochrome b-c1 complex subunit 2; CIV-COX1: mitochondrially encoded cytochrome *c* oxidase I. ***F***, Representative capillary western analyses and summary data showing that rmCHI significantly decreases Mff phosphorylation and increases Opa1 levels compared with sham controls. Metformin treatment restored Mff phosphorylation and increased Drp1 levels. Drp1: dynamin 1-like protein; Mff: mitochondrial fission factor; Opa1: optic atrophy 1; Mfn2: mitofusin-2; *p < 0.05 by one-way ANOVA.

Many studies have reported that concentrations of metformin in the millimolar range can decrease mitochondrial respiration by inhibiting Complex I activity (El-Mir et al., 2000; Detaille et al., 2002; Wheaton et al., 2014; Matsuzaki and Humphries, 2015; LaMoia and Shulman, 2021), although the physiological relevance of this inhibition remains controversial (Fontaine, 2018; Pecinová et al., 2019; LaMoia and Shulman, 2021). Consistent with previous results, we found that metformin concentrations higher than 2 mm significantly suppressed basal OCR in isolated hippocampal mitochondria (*F = *55.5, *p *<* *0.001; Fig. 4*A*). This suppression could not be recovered by the addition of ADP, but could be overcome by the addition of the uncoupler FCCP (Fig. 4*B*). As ADP requires an intact membrane potential to increase OCR, this data supports the premise that at high doses, metformin inhibits Complex I thereby disrupting the proton gradient of mitochondria.

We measured the concentration of metformin reached in the brain after intraperitoneal administration to assess the possibility that it might be inhibiting Complex I activity. Mice (*n* = 4) were intraperitoneally injected with 250 mg/kg metformin, and serum and brains were collected at 6 h for analysis of metformin using HPLC as described in Materials and Methods. This time point was chosen as it has been previously reported that metformin reaches its peak concentration in the brain at 6 h after administration, equivalent to the metformin concentration in the serum (Łabuzek et al., 2010). Representative chromatograms from processed cortical tissue extracts in the absence (blue) and presence (red) of spike-in metformin show that metformin eluted with a retention time of 6.59 min after sample injection (Fig. 4*C*). The serum concentration of metformin was calculated to be 24.03 ± 7.6 μm, comparable that reported in a previous study (Łabuzek et al., 2010). The concentrations of metformin in the hippocampus, cerebral cortex and the cerebellum ranged from 18 to 30 μm (Fig. 4*D*).

### Metformin and mitochondrial signaling pathways after rmCHI

Metformin has been demonstrated to increase mitochondrial biogenesis and alter mitochondrial dynamics (Kukidome et al., 2006; Wheaton et al., 2014; Jin et al., 2016; Izzo et al., 2017; Karise et al., 2019; Wang et al., 2019). We examined the expression levels of key components of the mitochondrial electron transport system by Capillary western analyses 2 h after the last injury. No significant differences in the immunoreactivity of ATP5a (ATP synthase subunit α; *F = *0.126, *p = *0.883), CIII core 2 (cytochrome b-c1 complex subunit 2; *F = *0.173, *p = *0.843), or CIV-COX1 (mitochondrially encoded cytochrome *c* oxidase I; *F = *2.387, *p = *0.134) were observed after rmCHI (rmCHI+Veh) or metformin treatment (rmCHI+Met) as compared with sham controls (*n* = 5/group), suggesting repeated injury did not increase mitochondria biogenesis (Fig. 4*E*). In contrast, when signaling pathways involved in regulating mitochondrial dynamics were interrogated, we detected an injury-related significant decrease in phosphorylated mitochondrial fission factor (Mff; *F = *7.717, *p = *0.008: sham vs rmCHI+Veh: *p = *0.014), and a significant increase in Opa1 levels (*F = *6.940, *p = *0.013; sham vs rmCHI+Veh: *p = *0.012), suggesting that rmCHI decreased mitochondrial fission (Fig. 4*F*). Metformin administration attenuated the injury-related decrease in Mff phosphorylation (rmCHI+Veh vs rmCHI+Met: *p = *0.018). Opa1 levels were found to be modestly decreased in response to metformin treatment, although this did not reach statistical significance (rmCHI+Veh vs rmCHI+Met: *p = *0.150; Fig. 4*F*). In addition, metformin treatment increased total Drp1 immunoreactivity in injured mice (*F = *6.248, *p = *0.015; rmCHI+Veh vs rmCHI+Met: *p = *0.040). No change in the total level of Mff (*F = *1.703, *p = *0.223) or mitofusin-2 (Mfn2; *F = *1.965, *p = *0.191) was observed as a result of rmCHI, or in response to metformin treatment.

### Metformin and vestibulomotor and motor function after rmCHI

**Figure 5.**
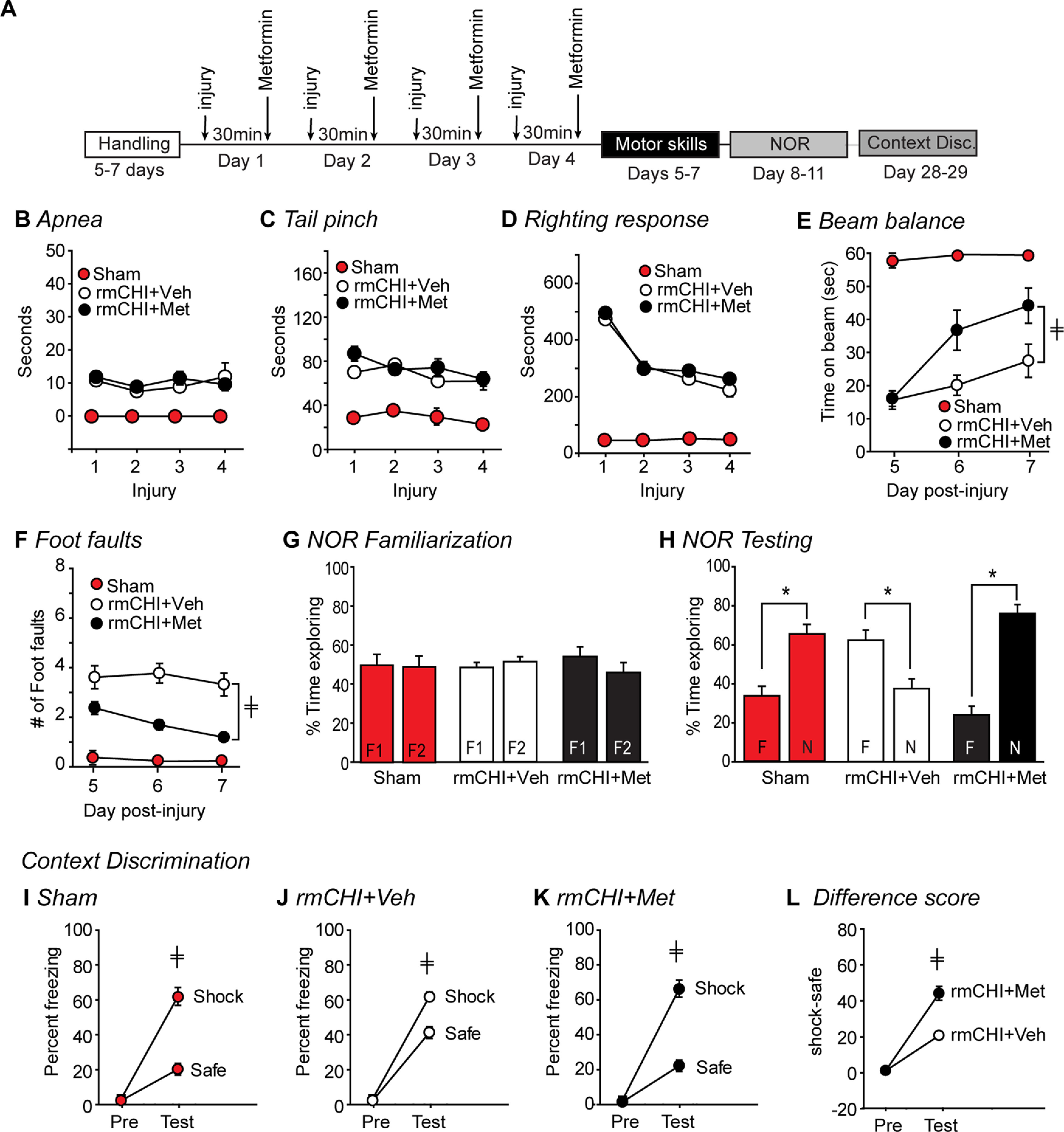
Postinjury administration of metformin after rmCHI reduced vestibulomotor, motor, and cognitive dysfunction. ***A***, Time line showing the experimental design. Sham, rmCHI+Veh, and rmCHI+Met (250 mg/kg) animals were tested immediately after injury for (***B***) duration of apnea, (***C***) suppression of tail pinch reflex, and (***D***) latency to regain righting response. Vestibulomotor and motor performances of sham, rmCHI+Veh, and rmCHI+Met mice were tested using the (***E***) beam balance and (***F***) foot fault (grid walking) tasks. On days 8–11, animals were tested for cognitive performance in the NOR task. ***G***, Percent time exploring the two identical objects (F1 and F2) used for familiarization in the NOR task. All groups equally explored both objects. ***H***, When tested for their object memory 24 h later, both sham and rmCHI+Met mice spent significantly more time exploring the novel object (N) rather than the familiar one (F), indicating intact recognition memory. rmCHI+Veh mice spent more time with the familiar object, an indication of perseveration. Performance of (***I***) sham, (***J***) rmCHI+Veh, and (***K***) rmCHI+Met in the context discrimination task. ***L***, Comparison of the freezing differentials between “shock” and “safe” contexts in the rmCHI+Veh versus rmCHI+Met mice. Data are presented as the mean ± SEM; ‡*p* < 0.05 by two-way repeated measures ANOVA; *p < 0.05 by paired *t* test.

We examined whether acute metformin treatment affected the vestibulomotor and motor dysfunctions resulting from rmCHI (*n* = 10/group). Vestibulomotor function was tested using the beam balance task, and motor function using the foot fault task. These tests were conducted for three consecutive days beginning 24 h after the last injury administration (Fig. 5*A*). A group of uninjured sham mice (*n* = 10) that were anesthetized once a day for 4 d were used as baseline controls for comparison. Acute neurologic measures were recorded after each sham or injury surgery. rmCHI caused a significant increase in postinjury apnea (*F = *19.201, *p* < 0.001; Fig. 5*B*), a suppression of the tail reflex (*F = *45.034, *p *<* *0.001; Fig. 5*C*), and increased latency to regain righting responses (interaction: *F = *9.103, *p *<* *0.001; Fig. 5*D*) when compared with sham controls. These acute neurologic changes did not differ between vehicle-treated and metformin-treated injured mice: duration of apnea (*p = *0.788); duration of suppression of pain reflexes (*p = *0.735); duration of suppression of righting responses (*p = *0.529). Beginning 24 h after the last sham or injury surgery, vestibulomotor and motor function was tested once a day for 3 d. Both vehicle- and metformin treated rmCHI groups (*n* = 10/group) displayed vestibulomotor dysfunction, as indicated by an inability to balance on a narrow beam (Fig. 5*E*). However, the rmCHI+Met treatment group showed significantly improved balance performance, indicated by longer latencies to fall off the balance beam when compared with vehicle-treated rmCHI animals (*F *=* *5.360, *p *=* *0.033; Fig. 5*E*). Similarly, although both injured groups made more foot faults than sham controls, motor function was improved in the rmCHI+Met group, as evidenced by a significant decrease in the number of foot faults compared with vehicle-treated injured mice (*F *=* *5.313, *p *=* *0.010; Fig. 5*F*).

### Metformin and memory after rmCHI

Beginning on day 8 postinjury, recognition memory was tested using the NOR task (Fig. 5*A*). Exploration of novelty (object or place) is an innate animal and human behavior. The results presented in Figure 5*G* show that all groups spent equivalent amounts of time exploring the two objects (F1 and F2) during familiarization, indicating there was no preexisting bias for the objects, or their positions in the testing chamber. Twenty-four hours later, one of the familiar objects was replaced with a novel object to test recognition memory. Uninjured sham mice spent more time exploring the novel object (N) than the familiar (F) one (*p *=* *0.014; Fig. 5*H*). In contrast, the rmCHI+Veh group spent more time exploring the familiar object, rather than the novel object (*p *=* *0.034) indicating impaired recognition memory. Acute postinjury administration of metformin to rmCHI mice improved recognition memory (*p *< 0.001; Fig. 5*H*), as indicated by animals spending significantly more time exploring the novel object. This performance by the metformin-treated rmCHI mice was comparable to that of sham-operated control mice.

Contextual fear discrimination task was administered beginning on day 28 postinjury (Fig. 5*A*). In this task, normal animals remain frozen for a longer duration of time in the chamber in which they had previously received a foot shock (shock chamber) as compared with a chamber in which they had not received a foot shock (safe chamber). Before foot shock, all groups displayed normal exploratory behaviors in both the safe and shock contexts, and did not assume a freezing posture (an indicator of fear). Figure 5*I* shows that sham-operated controls quickly learn to differentiate between the two similar but distinct contexts, displaying significantly more freezing behavior in the “shock” chamber than in the “safe” chamber after only a single training trial (*F *=* *50.307, *p *<* *0.001). Both the rmCHI+Veh (*F *=* *133.983, *p *<* *0.001; Fig. 5*J*) and rmCHI+Met mice (*F = *130.706, *p *<* *0.001; Fig. 5*K*) were also able to differentiate between the two contexts, albeit the difference in freezing behaviors exhibited between the “shock” and “safe” contexts were less than that seen in sham animals. When a discrimination difference score was calculated (shock freezing % – safe freezing %) and compared across injured groups, the metformin treated rmCHI animals showed a significantly improved ability to discriminate between the two contexts compared with vehicle-treated injured controls (*F *=* *21.966, *p *<* *0.001; Fig. 5*L*).

## Discussion

In the present study, we examined whether metformin administered after repeated mCHI (rmCHI) alters brain tissue bioenergetics, pathology, and functional outcome. Our study revealed three key findings: (1) rmCHI increased mitochondrial proton leak in the hippocampus 2 h after the last injury, an effect that was reversed by postinjury administration of metformin. In addition to attenuating injury-associated proton leak, metformin also significantly enhanced ATP-linked respiration in the hippocampus; (2) while rmCHI did not elicit visible tissue damage or loss of hippocampal synaptic proteins, it resulted in axonal damage in the corpus callosum and hippocampal commissure. These white matter disturbances were reduced in rmCHI mice that received acute metformin treatment; (3) metformin administration after each head impact significantly improved motor and memory function when tested days after the termination of the drug treatment. These results support the continued evaluation of metformin as a possible concussion therapeutic, and suggest that restoration of mitochondrial function may be an effective strategy to reduce inflammation and axonal injury.

The present study used a model of CHI to deliver repeated impacts separated by 24 h to mice. This paradigm was based on previous studies which demonstrated that a single mCHI was insufficient to produce reliable cognitive dysfunction (DeFord et al., 2002; Meehan et al., 2012). However, when injuries were separated by a 24 h delay, it was found that three to five repeated injuries produced significant hippocampal-dependent learning and memory dysfunction (DeFord et al., 2002; Meehan et al., 2012; Hylin et al., 2013). Our evaluation of acute neurologic responses after each injury revealed that, although apnea and recovery of pain reflexes remained consistent across injuries, the duration of suppression of righting response decreased after the first impact (Fig. 5*D*). While the reason for this decrease is not clear, a few studies have suggested that brain injury can activate preconditioning pathways that may offer protection against subsequent injuries (Bolton and Saatman, 2014). For example, it has been reported that the duration of apnea and the suppression of righting responses were maximal after the first impact, but decreased following additional impacts separated by either 24 or 48 h (Bolton and Saatman, 2014; Bolton Hall et al., 2016). In contrast, when the injuries are separated by 72 h, no preconditioning effects are evident on acute neurologic responses (Maynard et al., 2019). Although we did not observe any influence of postinjury metformin administration on the acute neurologic responses when the injuries were separated by 24 h, whether its administration can stimulate preconditioning pathways in more separated injuries remains to be examined. Further, it is important to note that while metformin was administered after each of the four injuries, its administration can be considered preinjury for the second, third, and fourth injuries. The reported half-life of metformin varies from study-to-study, ranging from 2 to 6 h in mice (Kinaan et al., 2015). As metformin administration occurred 23.5 h before each subsequent repeat injury, it is estimated that between 4 and 12 drug half-lives would have passed by the next injury, which calculates to >90% clearance of the drug. However, metformin signaling may persist well beyond the clearance of the drug. For example, Solskov and colleagues observed that a single dose of metformin can offer protection against coronary artery occlusion initiated 24 h after discontinutation of the treatment (Solskov et al., 2008). Thus, it is likely that a combination of both preinjury and postinjury effects of metformin contributed to the protection we observed.

A large body of evidence has shown that TBI increases intracellular calcium to pathologic concentrations, leading to calcium overload in mitochondria (Pandya et al., 2013; Vekaria et al., 2017). Mitochondrial calcium overload can cause proton leak, production of reactive oxygen species (ROS), and compromised oxidative phosphorylation, all of which have been observed following TBI (Griesbach et al., 2009; Pandya et al., 2009; Owens et al., 2013; Bano and Ankarcrona, 2018). Consistent with this, we observed that rmCHI caused a significant increase in acute proton leak within the hippocampus. This effect on proton leak was blunted in animals treated with metformin, and was associated with an increase in ATP-linked respiration. At present, it is not known how metformin exerts these protective effects, but several mechanisms are plausible (Kim et al., 2016, 2021; Chen et al., 2017; Deschemin et al., 2017; Xu et al., 2021). For example, it has been suggested that metformin may exert some of its protective effects through inhibition of mitochondrial complex 1, leading to an increase in the ADP: ATP ratio and AMPK activation (Owen et al., 2000). Although we observed that ≥2 mm metformin can inhibit complex 1 activity in isolated mitochondria, our analysis of tissue levels indicated that the brain concentration of metformin after intraperitoneal injection of 250 mg/kg (a dose previously used in a number studies and the present study) reached only 20–30 μm. Although we cannot rule out the possibility of limited (spatially and/or temporally) vascular disruption that could affect metformin concentration in a local area, the blood-brain barrier appears to remain intact after repeat mild TBI to rodents (DeFord et al., 2002; Kane et al., 2012; Meehan et al., 2012; Petraglia et al., 2014). Therefore, it is anticipated that the brain penetration of metformin is not appreciably changed as a result of the repeated injuries and is ∼1/100 of the dose needed to inhibit complex 1 activity *in vitro* (please see Fig. 4*A*,*D*). These findings are consistent with other studies which have questioned the *in vivo* relevance of Complex I inhibition in the action of metformin (for review, see Fontaine, 2018; Pecinová et al., 2019; LaMoia and Shulman, 2021). Although controversial, conformational changes in Complex I and metformin accumulation in mitochondria (because of its charge) have been proposed as mechanisms by which Complex I inhibition may remain plausible *in vivo* (Owen et al., 2000; Bridges et al., 2014).

Mitochondria are dynamic organelles which balance the processes of fusion and fission to meet the energy demands of the cell. Our capillary western analyses indicated that rmCHI decreased the phosphorylation of Mff, while simultaneously increasing the levels of Opa1. Mff is anchored within the outer mitochondrial membrane and is a component of the mitochondrial fission complex, serving as a receptor for Drp1 (Gandre-Babbe and van der Bliek, 2008; Otera et al., 2010). Mff phosphorylation by AMPK is thought to enhance mitochondrial fission (Toyama et al., 2016). Opa1 is a GTPase expressed on the inner mitochondrial membrane whose levels and activity correlate with the extent of mitochondrial fusion (Cipolat et al., 2004). Thus, the observed decrease in Mff phosphorylation and increase in Opa1 levels we observed after rmCHI suggest a shift in the dynamic balance of mitochondria toward fusion. Interestingly, metformin restored Mff phosphorylation and increased Drp1 levels, changes that suggest increased activation of pathways that mediate mitochondrial fission. As mitochondrial fission plays an important role in the removal of damaged mitochondria by autophagy (Zhang and Lin, 2016), this may underlie the reduced proton leak and increased ATP synthesis we observed in rmCHI mice treated with metformin.

One of the most interesting aspects of the present study is that postinjury metformin treatment improved cognitive function. After repeated CHI, mice displayed a significant recognition memory deficit, as well as a deficit in contextual discrimination, both processes that are dependent on the function of the hippocampus (Frankland et al., 1998; Broadbent et al., 2010; Cohen et al., 2013). These impairments were observed in the absence of overt neuronal loss or altered expression of synaptic proteins. However, we observed that rmCHI caused white matter damage in the corpus callosum and hippocampal commissure that was accompanied by an increase in astrocyte activation. Diffusion tensor imaging (DTI) studies of concussion subjects have routinely reported damage to major white matter tracts, including the corpus callosum, with the degree of axonal injury correlating with the severity of cognitive dysfunction (Bazarian et al., 2014; Oehr and Anderson, 2017; Zhu et al., 2019). Although we cannot conclusively link the observed white matter damage and astrocyte activation to the ensuing cognitive impairments, these pathologic changes were significantly reduced in injured mice treated with metformin. A few mechanisms that may underlie traumatic axonal injury have been proposed, including activation of the calcium-dependent protease calpain, decreased NAD levels, and ROS-mediated damage (Povlishock and Kontos, 1992; Kampfl et al., 1997; Büki et al., 1999; Bains and Hall, 2012; Maynard et al., 2019, 2020). As metformin has been demonstrated to inhibit calpain, activate the NAD-dependent deactylase SIRT1, and has been repeatedly shown to reduce free radical production (Bonnefont-Rousselot et al., 2003; Yang et al., 2017; Cuyàs et al., 2018; Wang et al., 2020b), one or more of these mechanisms may contribute to the protections that we observed.

Although this is the first report to describe protective effects of metformin in a model of repeat mild TBI, it has been previously observed that metformin treatment was beneficial following moderate-to-severe TBI. For example, moderate-to-severe cortical impact injury in rats caused a persistent decrease in AMPK activity that was reversed by postinjury treatment with metformin. This reversal was associated with a reduction in newborn hippocampal neuron loss and an improvement in spatial memory performance (Hill et al., 2010). Using a weight drop model of brain injury, Tao et al., demonstrated that metformin reduced microglial activation and the expression of pro-inflammatory cytokines, effects attributed to inhibiting NF-ĸB and p38 MAPK signaling (Tao et al., 2018). A randomized clinical trial examining the safety and efficacy of metformin in severe TBI patients found that metformin treatment resulted in decreased serum levels of the astrocytic protein S100B, suggesting reduced neuronal injury (Taheri et al., 2019). Taken together, these data suggest that metformin can restore mitochondrial function, reduce inflammation, offer axonal protection, and improve cognitive outcome after TBI, and supports its testing in persons at risk for repeat concussions such as athletes participating in contact sports and military personnel. As our study was conducted using only male mice, and sex-dependent effects of metformin have been reported (Park et al., 2017; Krysiak et al., 2018, 2020; Ruddy et al., 2019), additional experiments will be required to assess the influence of metformin on repeat injury in females.

## Synthesis

Reviewing Editor: Laura Bradfield, University of Technology Sydney Decisions are customarily a result of the Reviewing Editor and the peer reviewers coming together and discussing their recommendations until a consensus is reached. When revisions are invited, a fact-based synthesis statement explaining their decision and outlining what is needed to prepare a revision will be listed below. The following reviewer(s) agreed to reveal their identity: C. Edward Dixon. Note: If this manuscript was transferred from JNeurosci and a decision was made to accept the manuscript without peer review, a brief statement to this effect will instead be what is listed below.

The reviewer of the original manuscript found that the authors have done a good job of addressing most of the queries. Please address the remaining request of the reviewer to add information to the discussion regarding the length of the effect of metaformin. I also ask that, as only male mice were used in the current study, could this please be stated in the abstract and a comment added to the discussion that the results may not generalise to females.

Reviewer #1

This manuscript describes a very comprehensive multi-mechanism evaluation of the therapeutic effects of metformin in a mouse model or repetitive mild TBI (rmTBI). Importantly, it examined the effects of metformin on many of the top theoretic mechanisms of TBI: axonal injury, synaptic damage, impaired metabolism, neurogenesis, and astrogliosis. Acute metformin treatment attenuated axonal pathologies, astrogliosis, elements of mitochondrial function, motor, and cognitive deficits. This is an excellent evaluation of a single dose of metformin. The previous concern regarding potential blood-brain barrier damage has been adequately addressed. However, the concern that the second-fourth mTBI are effectively pretreated with metformin was only partially addressed. The Authors rebut that given the half-life of metformin is only hours, the levels during the subsequent injuries were marginal. While true, this doesn–t take into account the persistent signaling and gene expression responses to metformin. For example, a metformin is reported to be cardioprotective 24 hours after administration (Solskov, et al, 2008). The Authors are requested to comment on this in the Discussion.
